# Myocardial scar sizing and characterization predicts ventricular arrhythmia in cardiac resynchronization therapy candidates

**DOI:** 10.1186/1532-429X-13-S1-P247

**Published:** 2011-02-02

**Authors:** José T Ortiz Pérez, Juan Fernández Armenta, Antonio Berruezo, Etelvino Silva, David Andreu, Naiara Calvo, Susana Prat, Lluis Mont Girbau, Josep Brugada

**Affiliations:** 1 Thorax Institute. Hospital Clinic Barcelona, Barcelona, Spain

## Background

There is not enough evidence to support the use of combined Implantable Cardiac Defibrillators (ICD) and Cardiac Resynchronization Therapy (CRT) devices in all CRT candidates. Stronger predictors of malignant ventricular arrhythmia (VA) and sudden cardiac death (SCD) are required. The purpose of this study was to assess the value of myocardial scar extension and scar characterization (core and border zone) to predict VA in CRT patients.

## Methods

A standard Contrast-enhanced Cardiac Magnetic Resonance (ce-CMR) was performed pre-implantation of CRT device in 78 cardiac heart failure patients (64±11 years) with ischemic and non-ischemic dilated cardiomyopathy. Systolic function was assessed by cine imaging. Myocardial scar was characterized by a standard 2D segmented gradient echo inversion-recovery sequence applied in sequential short axis slices. Total scar was expressed as percentage of LV mass and the core and border zone as percentage of total scar). Patients were followed every 6 months to look for appropriate ICD therapy episodes.

## Results

During a mean follow up of 25 months (percentile 25^th^-75^th^=15-34 months), appropriate therapy of ICD devices occurred in 11.5% of patients. The analysis showed that the percentage of total scar was a strong predictor of VA (ventricular fibrillation or ventricular tachycardia) needing ICD therapy (HR 1.096 [1.053 to 1.143], p <0.001). In a Cox regression model adjusted for systolic function, etiology and type of prevention, the predictive value of the size of the scar remained unchanged (HR 1.156 [1.063 to 1.257], p = 0.001). The percentage of border zone of the scar was also associated with the occurrence of VA (HR 1.063 [1.022 to 1.101], p <0.001). However, the proportion of core was not significant (p = 0.662). The area under the ROC curve of total scar to predict VA was 0.94, and a cut-off value of <16% of LV mass had a 100% negative predictive value to exclude VA during follow-up.

## Conclusions

In TRC candidates with dilated cardiomyopathy of any etiology the extent of myocardial scar and the proportion of border zone predict the occurrence of VA. Assessment of scar burden and scar characterization using ce-CMR may be helpful to decide when combined ICD/CRT devices are indicated among CRT candidates.

